# Evaluation and Management Outcomes and Burdens in Patients with Refractory Chronic Cough Referred for Behavioral Cough Suppression Therapy

**DOI:** 10.1007/s00408-021-00442-w

**Published:** 2021-04-05

**Authors:** Laurie J. Slovarp, Marie E. Jetté, Amanda I. Gillespie, Jane E. Reynolds, Julie M. Barkmeier-Kraemer

**Affiliations:** 1 School of Speech, Language, Hearing, & Occupational Sciences, University of Montana, 32 Campus Dr., Missoula, MT USA; 2 Department of Otolaryngology-Head and Neck Surgery, School of Medicine, University of Colorado, Anschutz Medical Campus, Aurora, CO USA; 3 Department of Otolaryngology, Emory University School of Medicine, Atlanta, GA USA; 4 Department of Otolaryngology, University of Utah School of Medicine, Salt Lake City, UT USA

**Keywords:** Chronic cough, Refractory chronic cough (RCC), Speech-language-pathology (SLP), Behavioral cough suppression therapy (BCST), Cost-effectiveness

## Abstract

**Purpose:**

The purpose of this study was to investigate the typical symptoms and medical management characteristics of adult patients with refractory chronic cough (RCC) who are referred to speech-language pathology (SLP) for behavioral cough suppression therapy (BCST) in order to estimate cost-effectiveness and efficiency of current practice patterns for this population.

**Methods:**

One hundred sixty-four (164) patients with RCC referred for BCST were surveyed. Patients completed an initial survey at BCST onset related to symptom pattern and prior treatment, including the Leicester Cough Questionnaire (LCQ). Every four to six weeks patients completed follow-up surveys to assess their response to BCST.

**Results:**

Mean age was 58 years (83.5% women). The majority of patients reported their cough began two or more years prior to BCST. Approximately half (49%) reported seeing four or more physicians (including primary care physicians) and being prescribed four or more medications (57%) prior to BCST. Medications targeting post-nasal drip (72%), reflux (70%), asthma (56%), and allergies (56%) were most commonly prescribed. BCST resulted in a clinically significant improvement in 70.1% of participants. The mean change in LCQ for those who improved with BCST was 6.61. Over half (58%) reported they were *quite satisfied* to *completely satisfied* with their treatment response. The average time from enrollment to study completion was 64 days.

**Conclusion:**

The results of this study suggest early intervention with BCST may be a cost-effective and efficient option for patients with RCC.

**Supplementary Information:**

The online version contains supplementary material available at 10.1007/s00408-021-00442-w.

## Introduction

Chronic cough, defined as cough lasting more than 8 weeks, is highly prevalent [1] disabling [2], and carries substantial economic burden. The multifactorial nature of chronic cough frequently does not allow identification of a single etiologic mechanism for the cough [3]. As such, patients are typically evaluated with a multitude of tests and treated by numerous healthcare professionals including primary care physicians and subspecialists in pulmonology, allergy, otolaryngology, gastroenterology, and speech-language pathology. The goal of evaluation across various disciplines is to determine an individual’s specific chronic cough phenotype, the most common of which are upper airway cough syndrome, cough-variant asthma, gastroesophageal reflux (GERD), and eosinophilic bronchitis [4, 5]. When each of these has been evaluated and found to be absent or adequately treated yet symptoms persist, a patient is considered to have refractory chronic cough (RCC). Evaluation for utility of behavioral cough suppression therapy (BCST)—also referred to in the literature as cough control therapy, cough suppression therapy, Physiotherapy and Speech-Language Therapy Intervention, among other similar variations [6–8]—typically occurs after completion of empiric treatment and testing for these common cough phenotypes [9–12].

BCST is administered by speech-language pathologists (SLP) sub-specialized in disorders of the larynx and upper airway. The goal of BCST is to use cognitive-behavioral techniques to override the cough reflex and, by so doing, improve cough control and reduce cough sensitivity. There is moderate evidence supporting its efficacy for improving quality of life, reducing cough severity and frequency, and decreasing cough sensitivity in patients with RCC [8, 13–15]. BCST is safe, improves cough outcomes in up to 87% of patients, is inexpensive and efficient (i.e., patients typically need no more than four treatment sessions) [16]. As such, a trial of BCST may be more cost-effective than, and as effective as, other common empiric medical treatments for RCC. This study describes survey data from 164 patients who underwent BCST and outlines their treatment course and quality of life outcomes.

## Methods

This survey study was approved by the University of Montana Institutional Review Board on March 05, 2015 (IRB#: 242-14). The data reported herein describe a subset of data gathered in a larger ongoing study looking at symptom and treatment patterns of patients with RCC. The current study pertains to treatment patterns and response to treatment specifically in patients with RCC treated with BCST. Data used in this paper were collected from June 2015 to June 2020.

### Setting and Participants

All data were collected via Health Insurance Portability and Accountability Act (HIPAA) -compliant Qualtrics survey software or a paper form. Participants were recruited by SLPs who regularly provide evidence-based BCST [16–18]. Participating SLPs were from thirteen clinics across six different states in the United States and one hospital clinic in Australia. All participants were referred for BCST, were at least 18 years old, had a complaint of cough for at least the past 8 weeks, were not current smokers, did not have a formal pulmonary diagnosis (e.g., chronic obstructive pulmonary disease, asthma confirmed with objective testing), and had not taken an ACE-inhibitor medication within two months of enrollment.

### Procedures

#### Survey Development

The survey consisted of four parts: (1) relevant medical history, (2) patient-reported symptoms, (3) questions related to prior treatment, and (4) the Leicester Cough Questionnaire (LCQ) [19, 20]. Due to an oversight, the initial survey was missing one question (“How many physicians have you seen for your cough?”). This question was added after the first 38 respondents. Given the primary purpose of this paper is to describe treatment patterns in patients with RCC who are referred for BCST, we report only the results related to relevant medical history, prior treatment, and response to BCST. This portion of the survey can be viewed Online in Appendix A.

#### Survey Administration

SLPs at participating clinics were given three options to recruit potential participants—an electronic tablet, a paper form with self-addressed and stamped envelope, or a recruitment flyer that contained a web address, a QR code, and a phone number that allowed patients to enroll on their own time. The majority of clinics opted for paper forms or flyers. All participants completed the survey within one day of completing their BCST evaluation session.

Following enrollment, participants were contacted by mail, phone, text, and/or e-mail, depending on their indicated preference, every four to six weeks to monitor change. The follow-up survey consisted of the LCQ and questions related to compliance with treatment, effectiveness of treatment, and overall satisfaction with cough status. Compliance was asked on a scale from 1 (*not very compliant*) to 4 (*very compliant*). Treatment effectiveness was asked on a scale from 1 (*not at all effective*) to 7 (*completely effective*). Overall satisfaction was asked on a scale from 1 (*Not at all satisfied. I’m not any better*) to 7 (*Completely satisfied. My cough is gone*). If the participant reported an improvement in cough status, they were asked what they thought contributed to the improvement (i.e., *medical treatment, BCST, both,* or *I’m not sure*). Follow-up data continued to be gathered every four to six weeks until the participants reported a satisfaction of at least 5 (*Quite satisfied*), or until they were no longer making progress or no longer wished to participate in the study.

### Data Analysis

Data analyses were performed with Statistical Package for the Social Sciences (SPSS) version 26 software. The paired *t* test was used to determine significance of change pre- and post-BCST. The Mann Whitney *U* test was used to assess difference in compliance between those who improved with BCST and those who did not. Multiple linear regression modeling was used to determine if demographic and medical history data predicted change in LCQ. Spearman’s correlation was used to determine the association between perceived treatment satisfaction and the LCQ. Alpha was set at 0.05. Data are reported as *N* (%).

## Results

A total of 211 patients referred for BCST completed the enrollment survey. Of these, 164 completed follow-up surveys.

### Demographics

One hundred thirty-seven (83.5%) of the respondents identified as female. Mean age of the sample was 58. The majority identified as either Caucasian (66%) or unknown/do not want to report (29%). Demographic data, including age by decade, are presented in Table 1.

**Table 1:** Demographic, smoking history, and number physicians seen prior to BCST of participants (*N* = 164)

Demographic and clinical characteristics	Frequency (%)
Gender	
Female	137 (83.5)
Male	27 (16.5)
Age	
18–29	4 (2.4)
30–39	17 (10.4)
40–49	21 (12.8)
50–59	34 (20.7)
60–69	50 (30.5)
70–79	27 (16.5)
80–89	6 (3.7)
Unreported	4 (2.4)
Race/Ethnicity	
Hispanic/Latino	4 (2.4)
Black/African American	0 (0)
White/Caucasian	108 (65.8)
Native American/Native Hawaiian	3 (1.8)
More than 1 race	1 (0.6)
Unreported/Unknown	48 (29.2)
History of smoking	
Yes	34 (20.7)
No	126 (76.8)
Unreported	4 (2.4)
Length of cough	
2–4 months	18 (11.0)
4–6 months	11 (6.7)
6–12 months	24 (14.6)
12–18 months	6 (3.7)
18–24 months	13 (7.9)
> 24 months	85 (51.8)
Number physicians seen	
1	18 (10.9)
2	18 (11.0)
3	29 (17.7)
4	25 (15.2)
5	16 (9.8)
6	7 (4.3)
7	1 (.6)
8	5 (3.0)
10	6 (3.7)
11	1 (.6)
12	2 (1.2)

### Relevant Medical History

#### Smoking

Thirty-four (21%) respondents reported a history of smoking, 28 (82%) of whom quit smoking over 10 years prior. Eighteen of the 33 who smoked (55%), smoked 10 years or fewer (Table 1).

#### Cough Length and Prior Treatment

**Fig. 1:**
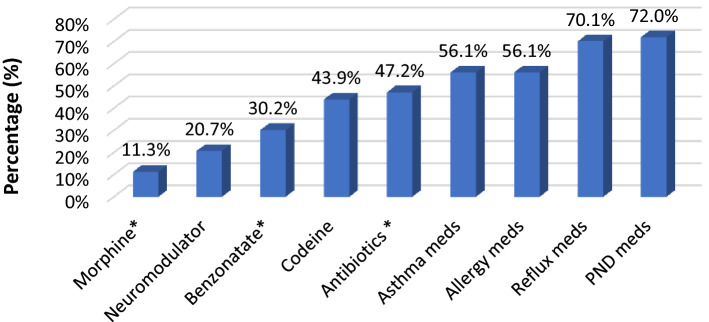
Medications prescribed to respondents prior to BCST referral. Neuromodulators include gabapentin, pregabalin, or amitriptyline. *Only the final survey version (*N* = 53) asked about morphine, antibiotics, and benzonatate. *PND* post-nasal drip

Length of cough was asked in a multiple-choice format with the longest option being “over 2 years”, which was the most common answer with 85 (52%) of the respondents. Only 29 (18%) reported a cough duration of six months or less (see Table 1). One hundred twenty-eight (128) respondents reported number of physicians seen, including generalists and specialists. The remaining left the question blank, reported they did not recall, or reported “too many to count”. Of the 128 who answered the question, 63 (49%) reported seeing four or more physicians prior to BCST. Only 18 (11%) reported seeing one physician, while 18 (11%) saw two physicians, and 29 (18%) saw three physicians. Data on cough length and number of physicians seen are included in Table 1. Ninety-three (57%) respondents reported being prescribed four or more medications prior to BCST. Over half reported being prescribed medications for post-nasal drip (*N* = 118, 72%), reflux (*N* = 115, 70%), asthma (*N* = 92, 56%), and allergies (*N* = 92, 56%). Distribution of prescribed medications is presented in Fig. 1.

### Response to BCST

Data from five participants were removed from the sample before analyzing response to BCST because it could not be determined if their improvement was due to BCST. One of these participants reported good BCST compliance and BCST effectiveness, but reported medical treatment was the only reason for improvement. The other four participants reported poor compliance to BCST and either contributed their improvement to medical treatment or reported “nothing has improved my cough.”

Mean total LCQ scores pre-and post-BCST for the remaining 159 respondents were 11.29 and 15.95, respectively. The distribution of LCQ change scores were approximately normally distributed. Between the 159 scores recorded, there were 154 unique values, further justifying treating LCQ change score as a continuous variable in a paired *t* test. The paired *t* test revealed a statistically significant increase of 4.66 (95% CI 4.02 to 5.30), *t*(158) = 14.39, *p* < 0.0005, *d* = 1.14. Each LCQ domain score also significantly improved (*p* < 0.0005 for each) and can be viewed in Table 2.

**Table 2:** Distribution of LCQ domain and total scores

	Pre-BCST		Post-BCST		Paired *t* test	
	Mean (SD)	Range	Mean (SD)	Range	LCQ Δ 95% CI	Sig
Physical	3.97 (1.17)	1.14–6.86	5.26 (1.18)	1.75–7.00	1.11 to 1.50	< .0005
Psychological	3.66 (1.16)	1.29–6.13	5.25 (1.65)	1.43–7.00	1.35 to 1.87	< .0005
Social	3.65 (1.37)	1.00–7.00	5.41 (1.59)	1.00–7.00	1.52 to 2.03	< .0005
Total	11.28 (3.18)	4.68–19.48	15.90 (4.23)	4.82–21.00	4.02 to 5.30	< .0005

*CI* confidence interval*, LCQ* Leicester Cough Questionnaire, *SD* standard deviation, *Δ* change

A conservative threshold of change in total LCQ score of at least 2.0 was set to indicate a clinically relevant improvement in cough, which is 0.7 points higher than the “minimum important difference” reported in the literature [21]. One hundred thirteen (70.1%) of the 159 respondents met this threshold. The mean change in LCQ for these 113 respondents far exceeded the 2.0 threshold at 6.61, resulting in a mean total LCQ score following BCST for these individuals of 17.74 out of a total possible score of 21.

Mean compliance score for those who improved and those who did not improve was 3.16 and 3.00, respectfully, an insignificant difference, *U* = 1626, *z* = −1.159, *p* = 0.246.

**Fig. 2:**
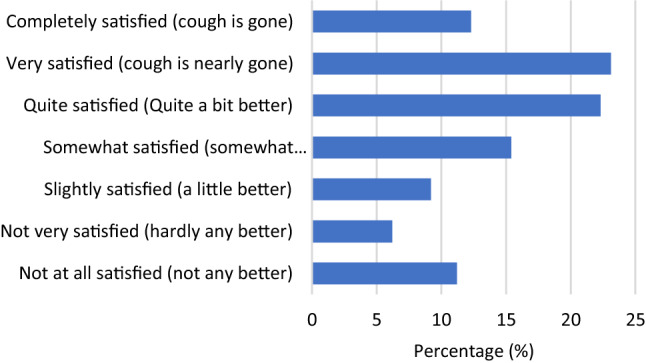
Distribution of satisfaction data

Spearman’s correlation on the entire sample (*n* = 164) revealed a strong positive correlation between patients’ perception of improvement and total LCQ scores, *r*_s_(133) = 0.746, *p* < 0.0005. Fifty eight percent (58%) reported they were quite satisfied (22.3%), very satisfied (23.1%), or completely satisfied (12.3%). Full distribution of satisfaction scores is shown in Fig. 2.

Multiple linear regression analysis was completed to develop a model for predicting LCQ change score from demographic and medical history data. There was moderate evidence that increased length of cough prior to BCST predicted lower LCQ change scores, (*F*(5,110) = 2.815, *p* = 0.02. No other variables had a significant effect on the model (see Table 3). The model was able to account for 15.2% of the variance in LCQ change score.

**Table 3:** Multiple regression results for LCQ change score

Predictor	*B*	Std. error	*t* value	*P*
Intercept	7.386	2.546	2.901	.004
History of smoking	− .198	.911	− .217	.828
Gender (male)	1.077	1.028	1.047	.297
Age	− .006	.030	− .192	.848
Cough (2–4 months)	− 3.159	1.946	− 1.623	.107
Cough (4–6 months)	− 1.871	1.483	− 1.262	.210
Cough (6–12 months)	− 2.518	2.145	− 1.174	.243
Cough (12–18 months)	− 5.550	1.866	− 2.974	.004*
Cough (18–24 months)	− 3.959	1.255	− 3.153	.002*
Number meds	.055	.163	.339	.735

Number of BCST sessions was not collected; however, the mean number of days between enrollment and final follow-up survey was 64. Given follow-up data requests were sent every four to six weeks until participants either reported a satisfaction score of at least 5 (i.e., *quite-a-bit satisfied, I’m quite-a-bit better*), or were no longer showing progress, the data suggests the majority of participants who improved with BCST did so within 5–9 weeks. LCQ and mean days from enrollment to final follow-up are presented in Table 4.

**Table 4:** Distribution of LCQ scores in participants who improved with BCST and those who did not improve with BCST

	Frequency (%)	Mean LCQ pre-BCST (SD)	Mean LCQ post-BCST (SD)	Mean Δ LCQ (SD)	Δ LCQ Range	Enrollment to follow-up (days)
Improved (Δ in LCQ > 2)	113 (71.0)	11.14 (2.79)	17.73 (2.70)	6.62 (3.17)	2.00 to 15.16	63.43 (43.83)
Did not improve (Δ in LCQ < 2)	46 (28.9)	11.66 (3.96)	11.58 (4.06)	− .066 (1.35)	− 3.14 to 1.86	65.95 (43.64)

*LCQ* Leicester Cough Questionnaire, *Δ* change, *SD* standard deviation

## Discussion

This prospective study adds to the growing body of literature highlighting the management difficulties of this disorder and the underutilization of BCST. The overarching goals in BCST are to (1) promote vocal hygiene techniques to reduce laryngeal irritation, and (2) train the patient to attend to and recognize the UTC sensation, and to prevent or interrupt the cough motor response by using volitional behavioral (cough suppression) techniques. The latter goal is achieved through principles of cognitive-behavioral therapy (CBT). Patients learn to implement a modified behavior (cough suppression techniques) in response to a trigger (cough stimulant) by training patients early on to recognize and attend to the urge-to-cough (UTC) sensation. If the patient can increase awareness of the UTC sensation and the circumstances under which it is triggered, and recognize that this sensation may indicate that a sensory threshold for producing the cough (motor response) has been met, then the patient can activate an alternative volitional motor response to suppress a cough [22, 23]. Exact techniques vary for training the patient to attend to their UTC sensation and to volitionally activate an alternative motor response using cough suppression strategies.

Despite the established efficacy of BCST, and its low-risk profile, most patients in this investigation reported seeking care for their cough from at least four physicians, and trialing at least six different medications intended to treat cough before being referred for BCST. At the time of enrollment in BCST, patients were still symptomatic, with low LCQ scores, indicating their cough negatively impacted quality of life. Following BCST, the total LCQ score increased on average over 4.6 points, indicating a significant and clinically meaningful improvement. Given the majority of participants included in this study reported having been prescribed four or more ineffective medications prior to BCST, these data suggest that BCST is at least as effective as some medical intervention for cough in the sample studied here. Given the average wait time to see a physician in the United States is approximately 24 days [24] and the most commonly prescribed empiric treatments for RCC (i.e., pharmaceutical treatments for rhinitis, GERD, or asthma) require a one-to-six-month trial period to determine effectiveness [25], the health and financial burden of such a protracted time to symptom resolution is significant.

Pharmaceutical treatments directly targeting cough hypersensitivity have been shown to be helpful in a proportion of patients with RCC; however, the data presented here suggests BCST is at least as, or more, effective, and with a much lower risk profile. Ryan et al. [26] is one of the few neuromodulator efficacy studies to include the LCQ as an outcome measure, allowing for direct cross-study comparison. Their placebo-controlled trial showed a mean change in LCQ of 2.5, which is nearly 2 full points lower than the mean LCQ change of 4.66 in the current study. Furthermore, 31% of the participants in Ryan et al.’s study experienced negative side effects. Our data also shows BCST to be superior to low dose morphine, which has been shown to reduce symptoms in approximately half of patients with RCC [8] with an average change in LCQ of 3.2.

The 2020 Medicare charge [27] for the most commonly prescribed tests for patients with RCC (i.e., chest and sinus CT, laryngoscopy, pulmonary function testing, allergy testing, swallow study, pH reflux testing) totals over $1200 (Table 5). With the average initial visit charge being $110, and the patients in this sample seeing on average at least 4 different physicians, $440 is spent on initial physician visits alone, totally over $1500 in tests and physician charges without counting the cost of repeat physician visits or medications (see Table 4). Conversely, the cost of one session of BCST is $81.20. Although we did not collect data on number of BCST sessions, prior studies indicate patients undergoing BCST typically receive no more than four sessions, for a total cost of $417.19. Further, and perhaps most importantly, these patients completed BCST *after* failing medical therapies, and only after BCST did they experience a meaningful improvement. Nearly 60% reported high satisfaction and symptom resolution with BCST, 29% of whom reported their cough was nearly or completely gone.

**Table 5:** Medicare HCPCS codes commonly billed in evaluation of refractory chronic cough

HCPCS CODE	Medical name	Non-facility price (private practice)	Facility price (hospital)
31575	Laryngoscopy without strobe	$125.95	$68.57
31579	Stroboscopy	$197.05	$123.07
31645	Bronchoscopy	$271.39	$152.66
70486	CT Sinus	$141.47	$141.47
71045	Chest X-Ray (1 view)	$25.98	$25.98
71046	Chest X-Ray (2 views)	$33.20	$33.20
71260	CT Chest	$199.21	$199.21
91038	PH/Impedance Testing	$449.32	$449.32
92507	Speech Therapy	$81.20	$81.20
92511	Nasopharyngoscopy	$114.76	$38.98
92520	Acoustic/Aerodynamic Measures	$82.28	$42.22
92524	Voice Evaluation	$92.39	$92.39
92610	Modified Barium Swallow Study	$89.14	$74.71
94010	Spirometry	$36.09	$36.09
94016	Analysis of Spirometry	$25.98	$25.98
94060	Bronchodilation challenge	$60.27	$60.27
94200	Included with Spirometry	$22.74	$22.74
94664	Teaching patient to use aerosol generating device	$16.96	$16.96
94726	Pulmonary function tests	$54.50	$54.50
95004	Allergy testing (40 pricks is typical)	$4.33	$4.33
95012	FENO for diagnosing asthma	$20.21	$20.21
95070	Bronchoprovocation challenge	$33.56	$33.56
99203	Level 3 new patient visit	$109.35	$77.23
99204	Level 4 new patient visit	$167.09	$132.09
99205	Level 5 new patient visit	$211.12	$172.51
99212	Level 2 established patient visit	$46.19	$26.35
99213	Level 3 established patient visit	$76.15	$52.33
99214	Level 4 established patient visit	$110.43	$80.48
99215	Level 5 established patient visit	$148.33	$113.68

Results of the current investigation support past BCST efficacy literature. In 1988, Blager [28] reported on four patients with refractory cough of presumed psychogenic origin. One patient underwent BCST with symptom resolution and cessation of cough suppression medications. In 2006, Vertigan and colleagues [17] published a prospective randomized placebo-controlled trial of four sessions of BCST (*n* = 47) compared to healthy lifestyle education training (*n* = 50). Eighty-eight percent of participants in the intervention group achieved a significant reduction in cough, compared to only 14% in the placebo group. Like participants in the current study, those in the Vertigan investigation underwent multiple diagnostic tests and medication trials prior to initiation of BCST. In 2017, Chamberlain Mitchell and colleagues [29] reported an improvement in LCQ of 3.4 in 34 patients following four sessions of BCST compared to improvement of only 1.53 in 41 control patients. Like the Vertigan study and ours, these patients had failed common empiric treatments prior to enrolling in BCST. Patients in the current study improved an average of 3.2 points more on the LCQ than the intervention group in the Chamberlain Mitchell investigation, adding further evidence to the strong efficacy of BCST. Taken together, the extant literature and the current investigation demonstrate that BCST is efficacious at reducing or eliminating cough, and cost-effective when compared to empiric medical treatments. Further, BCST can be initiated at any time in the diagnostic process without sacrificing accuracy of other diagnostic tests or empiric treatments. For example, patients could be offered BCST concurrently with empiric treatment for GERD, assuming they also have peptic symptoms (see, European Respiratory Guidelines [8]), which requires at least 4 weeks of medical management before symptom change [30].

The results of this survey highlight the need for several areas of future investigation. Randomized controlled trials would be beneficial for determining the role of first-line medical and behavioral and combined medical/behavioral therapies in improving objective measures of cough and quality of life. Based on the present data, we suggest consideration of early intervention with BCST is potentially more cost-effective and efficient for treating RCC than the conventional treatment model. An example of early intervention is offering BCST to a patient who visits their primary care physician because they have been coughing for 8 weeks following a resolved upper respiratory tract infection. Current standard of care is to first evaluate for red flags (i.e., hemoptysis, significantly productive cough, history of heavy smoking, prominent dyspnea at rest, hoarseness, systemic symptoms, difficulty swallowing, vomiting and recurrent pneumonia [12]) and obtain a chest x-ray, then prescribe empiric trials of proton pump inhibitors, inhalers, and/or nasal sprays, and finally refer to a pulmonologist and/or otolaryngologist if symptoms persist [31]. Future care could involve initiation of BCST at the time of empiric treatment (i.e., after ruling out red flags and normal chest x-ray). In some cases, early initiation of BCST might even expedite appropriate evaluation by specialists, as experienced SLPs with training in laryngeal and upper airway disorders may be able to recognize features of RCC that are consistent with more concerning pathologies (i.e., subglottic stenosis, tracheobronchomalacia, vocal fold lesions).

## Conclusion

The majority of patients who underwent BCST for RCC in this study experienced an improvement in their cough and quality of life in spite of previous extensive medical work up and treatment, suggesting that early intervention with BCST may be a more cost-effective and efficient option for patients with RCC.

## Supplementary Information

Below is the link to the electronic supplementary material.

Supplementary file1 (DOCX 20 kb)

Supplementary file2 (DOCX 16 kb)

## Data Availability

The first author holds all data and materials, which can be made available upon request.

## Abbreviations

BCST: Behavioral cough suppression therapy
GERD: Gastroesophageal reflux disease
IRB: Institutional Review Board
LCQ: Leicester Cough Questionnaire
RCC: Refractory chronic cough
SLP: Speech-language pathologists
