# Supplementary material for: Evaluation and Management Outcomes and Burdens in Patients with Refractory Chronic Cough Referred for Behavioral Cough Suppression Therapy
Source: Lung. 2021 Apr 5;199(3):263–71. doi: 10.1007/s00408-021-00442-w (PMC8203529; doi:10.1007/s00408-021-00442-w)
Supplement: Supplementary file 1 — Supplementary file1 (DOCX 20 kb) [file 408_2021_442_MOESM1_ESM.docx]

# Appendix A

##

**Relevant Medical History**

1. Do you have a history of smoking (beyond just trying it or smoking less than 12 months)?
2. How long did you smoke?

- Less than 5 years
- 5-10 years
- 11-15 years
- 16-20 years
- Over 20 years

1. How long ago did you quit smoking?

- Less than 1 year ago
- 1-5 years ago
- 6-10 years ago
- Over 10 years ago

1. How long ago did your cough start?

- 2-4 months
- 4-6 months
- 6-12 months
- 12-18 months
- 18-24 months
- Over 2 years

1. How many medical practitioners (physicians, nurse practitioners, physician assistants) have you seen for your cough (please estimate if you are not sure)?

## Which of the following medications have you tried and how long did you take each? (Circle your answer(s). Leave blank if not applicable.)

| Antibiotics | Less than 1 week | 1-2 weeks | 3-4 weeks | Over 4 weeks |
| --- | --- | --- | --- | --- |
| Medication for post-nasal drip | Less than 1 week | 1-2 weeks | 3-4 weeks | Over 4 weeks |
| Medication for reflux | Less than 1 week | 1-2 weeks | 3-4 weeks | Over 4 weeks |
| Medication for asthma (e.g., inhaled corticosteroid or rescue inhaler such as albuterol) | Less than 1 week | 1-2 weeks | 3-4 weeks | Over 4 weeks |
| Sinus spray or rinse | Less than 1 week | 1-2 weeks | 3-4 weeks | Over 4 weeks |
| Allergy medication | Less than 1 week | 1-2 weeks | 3-4 weeks | Over 4 weeks |
| Cough medicine with codeine | Less than 1 week | 1-2 weeks | 3-4 weeks | Over 4 weeks |
| Tessalon Perles (benzonatate) | Less than 1 week | 1-2 weeks | 3-4 weeks | Over 4 weeks |
| Neuromodulator (gabapentin (Neurontin), pregabalin (Lyrica), amitriptyline | Less than 1 week | 1-2 weeks | 3-4 weeks | Over 4 weeks |
| Morphine | Less than 1 week | 1-2 weeks | 3-4 weeks | Over 4 weeks |
| Other (please specify) | Less than 1 week | 1-2 weeks | 3-4 weeks | Over 4 weeks |

1. On a scale from 1-7, please rate the effectiveness of the medication in the past week in regards to your cough. (1 = not at all effective; 7 = completely effective)

## Leicester Cough Questionnaire (as described in the literature)
