# Supplementary material for: Evaluation and Management Outcomes and Burdens in Patients with Refractory Chronic Cough Referred for Behavioral Cough Suppression Therapy
Source: Lung. 2021 Apr 5;199(3):263–71. doi: 10.1007/s00408-021-00442-w (PMC8203529; doi:10.1007/s00408-021-00442-w)
Supplement: Supplementary file 2 — Supplementary file2 (DOCX 16 kb) [file 408_2021_442_MOESM2_ESM.docx]

**Description of speech-language pathology intervention for cough hypersensitivity syndrome: Behavioral cough suppression therapy (BCST)**

BCST is typically provided by a qualified speech-language pathologist and usually takes place across three or four sessions. BCST includes the following components: education, training in cough suppression strategies, reducing laryngeal irritation, and psychoeducational counseling.

- Education includes explaining the physiology of cough mechanisms and abnormal laryngeal movement, and providing a rationale for treatment. Education should include describing the role of neural plasticity and the anticipated reduction in laryngeal sensitivity in response to cough suppression that decreases the frequency of the urge to cough sensation and results in less coughing.
- Cough suppression strategies tailored to prevent or interrupt coughing, include breathing and laryngeal reposturing techniques that release laryngeal constriction, and promote efficient airflow during respiration and phonation. Cognitive-behavioral techniques are used to increase awareness of cough triggers and to attend to and recognize the urge-to-cough (UTC) sensation. The patient is trained to implement a cough-suppression strategy at the earliest sign of an UTC. Cough suppression strategies may include cough-control breathing strategies (e.g., open-throat breathing, pursed-lip breathing, exhaling through a cocktail straw), cough suppression swallowing (i.e., effortful swallow maneuver while pushing or pulling), as well as encouragement to sip water and use non-menthol lozenges. Patients are trained to complete cycles of suppression techniques daily outside of symptomatic periods to enhance generalization of strategy implementation at the first sign of an urge-to-cough sensation. Patients are trained to implement cough suppression strategies in functional communication contexts and during exposure to known cough triggers (e.g. in conversation, during a functional mobility task such as walking, or, given exposure to a safe household item that is a cough trigger for the patient).
- Reducing laryngeal irritation includes minimizing or eliminating laryngeal irritants including reflux, alcohol, and oral breathing. Patients are instructed in laryngeal hygienic strategies such as increasing hydration, using humidification, and reducing phonotraumatic behaviors.
- Psychoeducation includes counseling regarding the diagnosis and treatment of refractory chronic cough and cough hypersensitivity syndrome. Building a therapeutic alliance, exploring coping strategies, and drawing on the patient’s motivation and personal goals for recovery are key to the counseling process. In addition, outlining expectations for time and resource investment to maximize the benefit of therapy are addressed.
